# Cultural and relational factors in interpersonal distance regulation: evidence from a 2D screen-based task in Spain, Italy, and Japan

**DOI:** 10.3389/fpsyg.2026.1802960

**Published:** 2026-06-30

**Authors:** Giuseppe Iandolo, Oscar López de la Nieta, Carlos Benedicto, Seraphina Fong, Alessandro Carollo, Gianluca Esposito, Carolina Angel Ardiaca, Cristina Alonso Campuzano, Maria Alejandra Koeneke Hoenicka

**Affiliations:** 1 Department of Psychology, Universidad Europea de Madrid SLU, Madrid, Spain; 2 Dipartimento di Psicologia e Scienze Cognitive, Universita degli Studi di Trento, Rovereto, Italy; 3 Department of Psychology, Universidad Alfonso X el Sabio, Villanueva de la Cañada, Spain

**Keywords:** adult attachment dimensions, caregiving practices, cross-cultural psychology, interpersonal distance, screen-based task, visual perception

## Abstract

Interpersonal distance regulation varies across cultural contexts and individual relational orientations, yet its determinants in digitally mediated social environments remain insufficiently understood. This cross-sectional, cross-cultural study examined associations between early child-rearing practices (co-sleeping and co-bathing), adult attachment-related dimensions (anxiety and avoidance), perceptual sensitivity to visual context, and tolerance for interpersonal proximity in a controlled 2D screen-based task. A total of 305 adults from Spain, Italy, and Japan completed self-report measures, including the Experiences in Close Relationships scale, as well as three computerized tasks: the Ebbinghaus illusion, the Müller–Lyer illusion, and a personal-space judgment task involving various humanoid figures. Japanese participants reported higher prevalence and longer duration of co-sleeping and co-bathing, higher attachment-related avoidance, and greater tolerance for interpersonal closeness in the 2D task, whereas Spanish and Italian participants preferred larger interpersonal distances. Cross-cultural differences also emerged in susceptibility to visual illusions. Bivariate associations suggested links between early caregiving practices, attachment-related avoidance, perceptual sensitivity, and personal space preferences; however, these associations were substantially attenuated after adjusting for cultural context and demographic variables. Overall, the findings indicate that interpersonal distance regulation in screen-based contexts is more strongly associated with cultural norms than with early caregiving practices or adult attachment orientations, while perceptual processes intersect with social regulation in culturally patterned ways, suggesting that norms of interpersonal closeness may not translate directly from face-to-face interactions to screen-based settings.

## 1 Introduction

### 1.1 Personal space, attachment and cultural differences

Personal space, or interpersonal distance, refers to the minimum physical separation between individuals at which they feel comfortable (Sommer, 1969). Research on interpersonal distance is often examined through the lens of attachment theory, which explores how early relational experiences influence patterns of proximity and emotional connection in adulthood (Fraley and Roisman, 2019; Kaitz et al., 2004; Shrivastava and Burianova, 2014; Takano and Mogi, 2019). According to attachment theory, memories of physical proximity, comfort, emotional warmth, and protection serve as key indicators of attachment security (Bowlby, 1969, 1988). Empirical research supports this claim: mothers of infants characterized by lower attachment-related insecurity typically respond promptly and warmly to their distress (Leerkes and Crockenberg, 2003), individuals reporting lower attachment-related anxiety and avoidance tend to recall more positive, warmth-related early caregiving experiences (Bosmans et al., 2020), and the core functions of attachment (proximity-seeking and secure base) are rooted in early experiences of physical safety (Lahousen et al., 2019). When accompanied by caregiver support for exploration, these experiences contribute to the development of more coherent and secure Internal Working Models (IWMs) (Bretherton and Munholland, 2008; Rowe and Carnelley, 2005): mental representations of the self, others, and relational dynamics grounded in early interactions (Cassidy, 2013; Pietromonaco and Barrett, 2000; Kochanska and An, 2024).

During infancy, more secure IWMs provide emotional resources for self-regulation, even in the caregiver’s absence. Empirical research shows that sensitive parenting in early childhood supports the development of executive functions and self-regulatory capacities (Bernier et al., 2010), and that maternal meta-emotional knowledge facilitates effective emotion regulation even during separation (Grossmann et al., 2008). Additionally, more secure attachment representations support adaptive emotional regulation strategies across development (Zimmermann and Iwanski, 2015).

Attachment dimensions can also influence preferences for interpersonal distance. Research on attachment-related avoidance in childhood suggests that higher levels of avoidance are associated with a reduced expression of distress and a lower tendency to seek comfort from caregivers, even in stressful situations (Benoit, 2004; Main et al., 1985; Paquette et al., 2024). Attachment-related avoidance is characterized by a preference for emotional distance and self-reliance (Burgoon and Le Poire, 1999; Campbell et al., 2001; McCall, 2015; Lebert et al., 2024), reflecting discomfort with dependency and a tendency to manage challenges independently (Brennan et al., 1998; Cataldo et al., 2021; Fraley and Shaver, 1998). In adulthood, higher levels of attachment-related avoidance are generally associated with interpersonal distance and interactions involving strangers, as well as a tendency to underestimate potential interpersonal risks and overestimate one’s capacity for independent coping. This tendency can lead to inaccurate assessments of both distance and proximity, resulting in false negatives, that is, situations in which interpersonal proximity is not perceived as potentially uncomfortable or intrusive, even when it may be, due to an underestimation of threat or emotional relevance (Chun et al., 2015; Mikulincer and Shaver, 2019, 2023; Uccula et al., 2023).

In contrast, individuals with higher levels of attachment-related anxiety exhibit an intense need for proximity and heightened sensitivity to perceived threats of separation or rejection, leading to insecurity and an increased desire for closeness (Cassidy and Berlin, 1994; Brennan et al., 1998; Fraley and Shaver, 1998). In adulthood, higher levels of attachment-related anxiety are associated with hypervigilance and persistent efforts to maintain closeness, often linked to concerns about abandonment (Collins and Feeney, 2000; Liu and Ma, 2019). Accordingly, attachment-related anxiety is associated with greater discomfort during physical separation and in interactions with unfamiliar individuals, often amplifying efforts to seek attention and reassurance (Main and Solomon, 1986; Mikulincer and Shaver, 2023).

Cultural norms further shape both attachment patterns and interpersonal distance regulation, making them a central focus in cross-cultural psychology. Research suggests cultural differences in attachment dimensions, with Asian cultures exhibiting higher levels of attachment avoidance, while Mediterranean (Spanish and Italian) cultures show higher levels of attachment anxiety (Bassi et al., 2022; Koeneke-Hoenicka et al., 2022; López-de-la-Nieta et al., 2021; Nakao and Kato, 2004). Cultural norms also influence preferences for interpersonal distance (Perry et al., 2013). For example, Hispanic and Mediterranean cultures (South American, Southern and Eastern Europeans) generally favor closer physical proximity and show greater tolerance for interpersonal closeness. In contrast, North American, Northern European, and Asian cultures tend to prefer greater physical distance, where proximity may elicit discomfort (Beaulieu, 2004; Sicorello et al., 2018; Sorokowska et al., 2017; Sussman and Rosenfeld, 1982).

Within this framework, the present study examines how interpersonal proximity is regulated in a standardized 2D screen-based context across Spanish, Italian, and Japanese participants. The study brings together sociocultural context, adult attachment-related dimensions, retrospective caregiving experiences, and sensitivity to visual context. It aims to clarify which of these factors are most closely associated with comfort with approaching social stimuli in a low-emotional digital setting. Most research on attachment and interpersonal distance has focused on interactions with attachment figures, such as caregivers and romantic partners (Main et al., 1985; Mikulincer and Shaver, 2023). These studies emphasize how attachment dimensions influence preferences for closeness or distance with individuals who provide emotional security. However, some studies have also examined proximity regulation in the context of interactions with strangers, using stimuli unrelated to attachment figures, thereby introducing a distinct dimension to the analysis of relational closeness and personal space (Cartaud et al., 2020; Kaitz et al., 2010; McCall and Singer, 2015). The choice of experimental paradigm also plays a role: some studies employ stop-distance tasks or measure seating preferences in front of an unfamiliar, physically present interviewer (Hautle et al., 2024; Kaitz et al., 2004; Maier et al., 2020; Sorokowska et al., 2017), while others use virtual paradigms to assess interpersonal distance, closeness, and comfort (Han et al., 2023; Kroczek et al., 2020; Riem et al., 2019). Notably, studies employing virtual interpersonal distance paradigms, using screen-based or immersive tasks, do not support the assumption that virtual and real-life presentations yield equivalent results (Karakoc et al., 2025; Kroczek et al., 2020; Riem et al., 2019). Rather, virtual environments provide a controlled, ethical, and replicable framework for studying interpersonal distance and social comfort, even if their ecological validity remains distinct from real-world interactions. Accordingly, the present study draws on embodied interpersonal-distance research as a conceptual foundation, while treating the 2D task as a standardized, low-emotional measure of visual comfort with approaching social stimuli rather than as a direct equivalent of real-life interpersonal distance regulation.

Although interactions with strangers do not directly activate the attachment system, they may still reflect broader proximity-regulation strategies linked to individual differences in attachment-related anxiety and avoidance. For example, individuals with higher attachment-related anxiety may seek closeness even with strangers, while those with higher attachment-avoidance scores may show tolerance or indifference toward such closeness, consistent with their general tendency to downplay interpersonal intimacy (Collins and Feeney, 2000; Feeney, 2008; Szeifert et al., 2025). Research indicates that individuals with higher attachment-related avoidance typically maintain larger interpersonal distances, especially in emotionally vulnerable contexts; however, they may tolerate closer proximity when interactions lack emotional intimacy or personal salience, including in virtual or screen-based interpersonal-distance paradigms. In contrast, individuals with higher attachment-related anxiety may display contradictory behaviors, at times allowing intrusions into their personal space due to fear of rejection (Akbarian et al., 2022; Bar-Haim et al., 2002).

Consistent with this pattern, when interactions lack emotional intimacy or personal salience, individuals may rely on deactivation or minimization strategies to regulate discomfort (Mikulincer and Shaver, 2008, 2019; Sheinbaum et al., 2015). This allows them to adapt to situations that require reduced personal space, such as public transportation.

### 1.2 Child rearing practices, attachment and cultural differences

Child-rearing practices encompass the beliefs, values, and behaviors that shape how parents raise their children, including fundamental activities such as feeding, playing, communicating, and establishing daily routines (Bornstein, 2019; Gómez, 2019; Bornstein et al., 1992, 2008a, 2008b, 2012; Tudge, 2008). Certain child-rearing practices, such as co-sleeping and close physical touch, play a crucial role in shaping early attachment experiences and the perception of personal space, as they promote emotional security and physiological synchrony between caregiver and infant (Feldman et al., 2002; Feldman, 2012; Feldman and Eidelman, 2003; Lerner et al., 2020). Co-sleeping refers to parents sharing a sleeping space with their children, either by bed-sharing or room-sharing (Mileva-Seitz et al., 2017). In Eastern cultures like Japan, co-sleeping is widely practiced and associated with fostering emotional closeness and interdependence in early caregiver–child relationships (Esposito et al., 2015; Yang and Hahn, 2002).

Similarly, co-bathing, though less frequently studied than other child-rearing practices, is considered a normative and culturally meaningful activity in Japan, where it reinforces familial intimacy and attachment (Hallers-Haalboom et al., 2016). In contrast, in many European societies, co-bathing tends to be regarded as a private behavior and is less commonly practiced in later stages of infancy (Gardiner and Kosmitzki, 2005; Richman et al., 1988). However, to date, no studies have specifically explored co-bathing within Mediterranean populations in relation to attachment dimensions or perceptions of personal space.

In cultural contexts where co-sleeping and co-bathing are more common (e.g., several East Asian settings), socialization tends to emphasize emotional interdependence and comfort with close physical proximity, reflected in the high prevalence of shared sleeping arrangements and parental involvement at bedtime. By contrast, in contexts where these behaviors are less frequent, norms more often prioritize independence and personal space, encouraging solitary sleep and greater physical autonomy from early childhood (Ball et al., 2000; Mindell et al., 2010a, 2010b). Accordingly, co-sleeping and co-bathing were not conceptualized as isolated developmental variables independent of culture, but rather as caregiving practices embedded within broader sociocultural systems.

Individuals from collectivist cultures, such as Japan, where co-sleeping and co-bathing are common, may feel more comfortable with interpersonal closeness when constrained by external circumstances. In contrast, individuals from Mediterranean or Western cultures, where child-rearing emphasizes independence, may prefer to maintain greater interpersonal distance in the presence of people with whom they do not feel emotionally close (Mikulincer and Shaver, 2003, 2008, 2019, 2023; Sheinbaum et al., 2015).

### 1.3 From illusion to interaction: perception and proximity in 2D screen-based tasks

Optical illusions provide a powerful lens through which to examine biases in implicit visual processing (Tryon, 2014; Weiss et al., 2002). Among these, the Ebbinghaus and Müller-Lyer illusions are widely used to assess individuals’ susceptibility to visual distortions and contextual influences (Amir and Firestone, 2025). These illusions exploit perceptual cues that mislead judgments of size or length, making them ideal tools for probing the extent to which observers rely on holistic versus local information. Importantly, susceptibility to such illusions reflects not a fixed perceptual style, but rather an individual’s general sensitivity to contextual cues in visual perception (Chamberlain et al., 2017; Witkin et al., 1962).

A growing body of research has documented individual and cultural differences in illusion susceptibility, shaped by variables such as age, gender, developmental profile, neurodiversity, and psychopathology (Chouinard et al., 2016, 2019; Doherty et al., 2010; Van der Hallen et al., 2015). Cross-cultural studies consistently demonstrate that East Asian collectivist cultures tend to engage in holistic information processing, integrating contextual and background elements into perception and cognition. In contrast, Western individualist cultures exhibit a more analytic perceptual style, attending to focal objects and specific details within a scene (Nisbett, 2006; Masuda and Nisbett, 2001, 2006; Miyamoto et al., 2006; Senzaki et al., 2016; Markus and Kitayama, 1991; Li et al., 2025; Šašinková et al., 2023). These perceptual tendencies are rooted in culturally embedded norms, socialization practices, and environmental demands (Uskul et al., 2008; Miyamoto et al., 2006).

Recent research shows that such perceptual variations extend into the domain of interpersonal behavior, especially in digitally mediated or culturally diverse contexts, where traditional social cues are transformed (Chen-Xia et al., 2023; Goyal and de Keersmaecker, 2022; Norenzayan and Heine, 2005). As such, incorporating measures of visual bias into social interaction research may offer a deeper understanding of how culture shapes subjective interpretation in an increasingly globalized and digitally mediated world.

In the present study, the Ebbinghaus and Müller-Lyer illusions were embedded within a 2D screen-based task. These measures were used to examine whether contextual sensitivity in visual processing parallels individual differences in interpersonal distance regulation during a subsequent 2D screen-based task. The 2D screen-based Virtual Interpersonal Distance Paradigm provided a controlled environment for examining comfort with visual interpersonal proximity, without the emotional intensity of face-to-face interactions (Veranic et al., 2025). Prior research has shown that digital environments often result in lower emotional synchrony and empathic engagement (Ortiz Sobrino et al., 2019).

Importantly, interpersonal distance regulation may not operate identically across face-to-face and screen-mediated contexts. Because 2D screen-based task reduce the emotional intensity and embodied cues of real-life encounters, cultural norms associated with interpersonal closeness may be expressed differently in digitally mediated settings. Furthermore, individuals with higher levels of attachment-related avoidance or social anxiety often prefer screen-mediated interactions due to their reduced affective demands (D’Arienzo et al., 2019; Nelson, 2024; Read et al., 2018). Prolonged reliance on digital communication has been linked to diminished nonverbal decoding skills and more superficial forms of empathy, potentially impairing social bonding and emotional regulation (Čekić, 2025; Nguyen et al., 2022; Ruben et al., 2021). However, the present 2D screen-based task was not designed to assess emotional regulation directly, but rather to examine comfort with visual interpersonal proximity in a controlled, low-emotional context. By linking perceptual and social-cognitive domains, this approach highlights how biases in low-level visual processing may relate to comfort with interpersonal proximity in controlled screen-based interactions.

The aim of the present study was to examine associations among cultural context, retrospective child-rearing experiences, adult attachment-related anxiety and avoidance, and perceptual sensitivity to visual context were associated with comfort with interpersonal proximity in a 2D screen based task among Mediterranean (Spanish and Italian) and Japanese participants. To address this aim, the study adopted a mixed, cross-sectional and cross-cultural design combining self-reported questionnaires and experimental 2D screen-based tasks as complementary measures.

The study adopted a social-personality approach to adult attachment, conceptualizing attachment-related anxiety and avoidance as continuous individual-difference dimensions assessed via self-report measures. In line with contemporary attachment research, the study does not rely on categorical attachment classifications, nor did it aim to infer early attachment styles or developmental attachment representations. Rather, attachment dimensions were examined as relational orientations in adulthood that may be associated with interpersonal distance regulation, particularly in low-emotional, screen-based social contexts. References to early caregiving experiences were therefore treated as retrospective and biographical correlates, rather than as direct indicators of developmental attachment classifications.

To address this aim, three hypotheses guided the study:

*H1*: Engagement in early child-rearing practices, such as co-bathing and co-sleeping, is expected to be associated with lower self-reported levels of attachment-related avoidance in adulthood.

*H2*: Child-rearing practices, such as co-bathing and co-sleeping, are expected to be associated with greater tolerance for interpersonal closeness in a 2D screen-based experimental task involving various humanoid figures (a robot, a boy, a girl, an adult man, an adult woman, and an elderly woman).

*H3*: Higher levels of attachment-related avoidance are expected to be associated with greater tolerance to closeness in a 2D screen-based experimental task involving various humanoid figures (a robot, a boy, a girl, an adult man, an adult woman, and an elderly woman).

Conceptually, the present study adopted an integrative and exploratory approach, examining interpersonal distance regulation across multiple levels of analysis. At a sociocultural level, country was used as a pragmatic proxy for broader cultural norms related to interpersonal space and social interaction, while acknowledging that country-level differences may also reflect other contextual and demographic influences. At an individual level, adult attachment dimensions were considered as relational orientations that may be associated with comfort with interpersonal proximity. At a biographical level, retrospective reports of early child-rearing practices were examined as contextual correlates. Finally, at a perceptual level, susceptibility to visual illusions was included as an index of sensitivity to contextual visual information. Rather than proposing a single causal pathway, the study explored whether and how these levels converge or diverge in a controlled, screen-based interpersonal distance task, thereby contributing to a more nuanced understanding of personal space regulation in digitally mediated social contexts.

## 2 Methods

### 2.1 Participants

Participants consisted of 305 university students (Table 1): 32.78% from Madrid, Spain (*n* = 100), 27.87% from Trento, Italy (*n* = 85), and 39.34% from Nagasaki, Japan (*n* = 120). The overall sample included 52.5% males (*n* = 160) and 47.5% females (*n* = 145), with a mean age of 22.72 years (SD = 5.28). All participants completed a sociodemographic questionnaire that included questions about prior psychological or psychiatric diagnoses, as well as child-rearing practices during infancy. Participants did not report a history of psychological or psychiatric diagnosis. Informed consent was obtained from all participants prior to data collection. The study was approved by the Ethics Committee of Hospital Universitario de Getafe, Madrid, Spain (protocol code 2017A0517, and date of approval: 9 March 2017).

**Table 1:** Descriptive statistics of the participant sample.

Country/Sample	**Age**				**Gender**		**Total**
	Min	Max	Mean	SD	Female	Male	
Spain (Psychology and medicine students)	17	54	24.07	7.88	50 (50%)	50 (50%)	100
Italy (Psychology students)	18	34	22.76	3.17	59 (69%)	26 (31%)	85
Japan (Medicine students)	19	37	21.55	3.08	36 (30%)	84 (70%)	120
Total	17	54	22.72	5.28	145 (47.5%)	160 (52.5%)	305

To describe demographic differences in the present sample across countries, two non-parametric analyses were conducted. A Chi-Square Test on gender distribution revealed significant differences between countries (*χ*^2^ = 31.35; *p* < 0.01), indicating that gender representation varied significantly within the current study sample. Specifically, Spain had an equal distribution of female and male participants (50 each), Italy had a higher proportion of females (59 females vs. 26 males), and Japan displayed the opposite pattern, with more male participants (84 males vs. 36 females).

A Kruskal-Wallis test comparing age distributions also revealed significant differences (*H* = 9.20; *p* = 0.01), indicating that participant ages were not evenly distributed across the three countries (Table 1), with Spain showing a wider age range than the Italian and Japanese samples.

### 2.2 Measures

The study’s instruments consisted of three measures: a brief sociodemographic and clinical-screening questionnaire, the Experience in Close Relationship Scale - ECR (Brennan et al., 1998), and three experimental tasks (Ebbinghaus size comparison task, Müller-Lyer size comparison task, and Personal Space visual task).

The sociodemographic and clinical-screening questionnaire collected information on participants’ age, childhood rearing practices (co-sleep and co-bath), and previous clinical psychological or psychiatric diagnoses. Participants reporting previous diagnoses were excluded to control for potential confounding effects on attachment and perceptual measures (S1, Sociodemographic and clinical-screening questionnaire, Supplementary material S1 for the full questionnaire). Child-rearing practices were assessed retrospectively through self-report items asking participants whether co-sleeping and co-bathing occurred during childhood (binary indicators: yes/no), as well as the approximate age at which these practices ended (duration indicators). The binary variables were intended to capture the presence or absence of these caregiving practices within the participant’s developmental context, whereas duration variables were included to estimate the temporal extent of early bodily proximity experiences.

The Experience in Close Relationships Scale - ECR (Brennan et al., 1998) is a gold standard questionnaire for assessing adult attachment. It measures two dimensions of attachment, with 18 items assessing the dimension “attachment-related anxiety” and 18 items assessing the factor “attachment-related avoidance” (Cronbach’s *α* Anxiety 0.94; Cronbach’s α Avoidance: 0.91; Brennan et al., 1998) through 36 items on a 7-point Likert scale ranging from (1) “Strongly Disagree” to (7) “Strongly Agree.” Scores on these dimensions are conceptualized as continuous indicators of individual differences in adult attachment, capturing relative levels of attachment-related anxiety and avoidance. Although historically described in relation to prototypical attachment styles, the present study adopts a dimensional approach and does not rely on categorical classifications (Bartholomew, 1990; Bartholomew and Horowitz, 1991; Brennan et al., 1998; Fraley and Shaver, 1998; Gillath et al., 2016; Simpson and Rholes, 1998). To ensure cultural validity, the study used culturally adapted ECR versions: the ECR-S for Spain (α _Anxiety_ = 0.85; α _Avoidance_ = 0.87; Alonso-Arbiol et al., 2007), the ECR-I for the Italian sample (α _Anxiety_ = 0.89; α _Avoidance_ = 0.89; Busonera et al., 2014; Picardi et al., 2002) and the ECR-J for the Japanese sample (α _Anxiety_ = 0.87; α _Avoidance_ = 0.91; Nakao and Kato, 2004).

The study employed three computer-based experimental tasks, administered on a 14-inch Windows laptop: the Ebbinghaus size comparison task, the Müller-Lyer line length comparison task, and the Personal Space Visual Task. These tasks were developed and executed using MATLAB R2017a (MathWorks), Unity 2017.1, and Psychtoolbox-3 (v3.0.14). They were programmed to assess participants’ susceptibility to visual illusions and their comfort with interpersonal proximity, thereby providing a controlled framework linking perceptual processing to social distance preferences.

**Figure 1:**
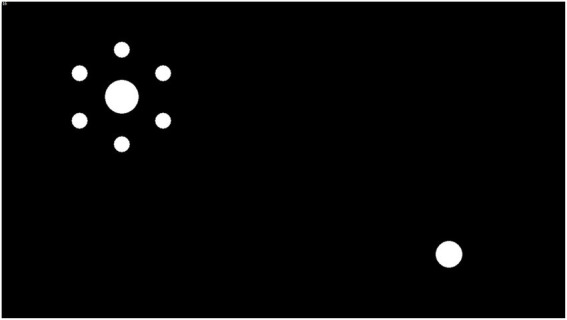
Experimental task for Ebbinghaus size comparison task. Participants adjusted the size of a comparison circle to match a prototype circle surrounded by contextual circles that were either larger or smaller, creating the Ebbinghaus illusion. The task assessed susceptibility to contextual size distortion by measuring discrepancies between adjusted and target circle sizes.

The Ebbinghaus size comparison task was based on the Ebbinghaus illusion, which demonstrates how contextual contrast in size can distort perceptual judgment. In this task, participants adjusted the size of a comparison circle (43 pixels in diameter) located in one corner of the screen to match a prototype circle surrounded by elements that were either 20% larger or smaller (Figure 1).

Each condition was presented across four trials per corner, resulting in a total of 16 experimental trials. The Ebbinghaus Accuracy Index (EAI) was calculated as the signed mean difference between the adjusted and reference circle sizes across trials. Negative values indicated underestimation, while positive values reflected overestimation relative to the target size. This measure was designed to capture participants’ accuracy in matching the prototype circle, thereby reflecting their susceptibility to contextual distortions.

**Figure 2:**
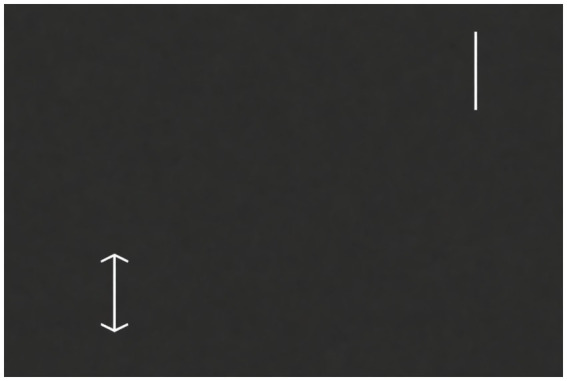
Experimental task for Müller-Lyer length comparison task. In this screen-based task, participants adjusted the length of a test line to match a prototype line flanked by inward- or outward-facing arrowheads, which created the Müller-Lyer illusion. The task assessed susceptibility to contextual visual distortion by measuring the discrepancy between adjusted and target line lengths.

The Müller-Lyer line length comparison task was based on the Müller-Lyer illusion, in which two lines of identical length appear unequal due to either inward- or outward-pointing arrowheads (Gregory, 1966; Howe and Purves, 2005). Participants adjusted the length of a test line (104 pixels in length) to match a prototype line flanked by inward- or outward-facing arrowheads that was either 20% longer or shorter (Figure 2).

As in the Ebbinghaus task, the reference line appeared in four trials per corner, yielding 16 trials in total. The Müller-Lyer Accuracy Index (MAI) was computed as the mean signed error between the adjusted and target line lengths, with higher values indicating greater distortion and susceptibility to the illusion.

The Ebbinghaus and Müller-Lyer screen-based tasks served as baseline measures of susceptibility to visual biases, offering insight into how such biases are related to spatial judgments and the relationship between visual processing style and comfort with interpersonal distance in 2D virtual environments.

**Figure 3:**
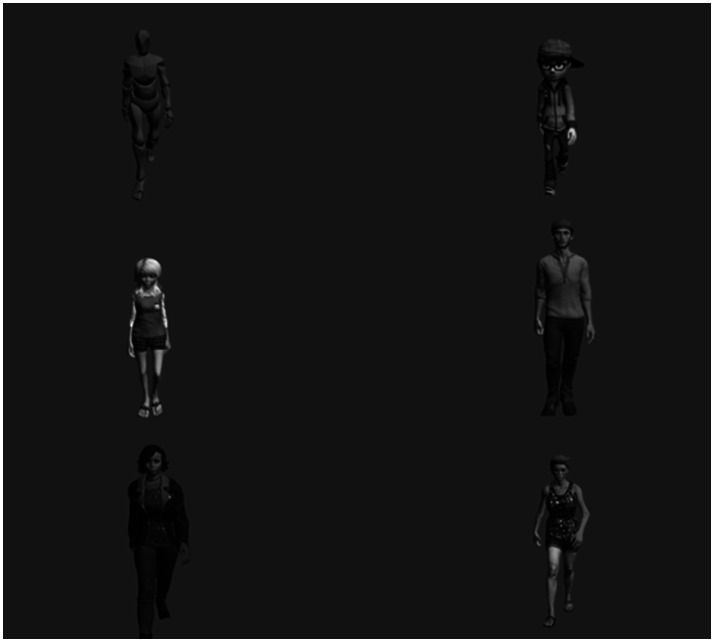
Humanoid avatars in the Personal Space Visual Task. The task included six two-dimensional approaching avatars: a robot, a boy, a girl, an adult man, an adult woman, and an elderly woman. Participants stopped each avatar when it was perceived as being “too close,” and the remaining distance served as an index of comfort with interpersonal proximity in the screen-based task.

The Personal Space Visual Task assessed participants’ comfort with interpersonal proximity through a series of two-dimensional, screen-based virtual interactions involving six approaching humanoid avatars: a robot, a boy, a girl, an adult man, an adult woman, and an elderly woman (Figure 3).

Each avatar was presented at two gait speeds, slow (2.4 cm/s) and fast (4.8 cm/s), simulating a naturalistic walking pace in a virtual environment. The avatars approached the participant along a linear path with a maximum travel distance of 10.36 virtual units (equivalent to 1,036 pixels on screen). Participants initiated and stopped the avatars’ movement by pressing the space bar, indicating the moment at which the avatar was perceived as being “too close.” The primary variable of interest was the remaining distance between the avatar and the participant at the moment of discomfort, which served as an index of personal space regulation.

The avatar appeared walking progressively toward the observer from depth, approaching in a straight line. Each of the six avatars was presented once at the slow speed and once at the fast speed, yielding 12 trials in total. The Personal Space Distance Remaining Mean Index (PS-DR_m) was computed as the mean remaining distance between the avatar and the observer across the 12 trials, with higher values reflecting greater interpersonal distance preference.

### 2.3 Procedure

Participants were individually assessed at university facilities in Madrid (Spain), the Trento (Italy), and Nagasaki (Japan). Data were collected in individual, in-person sessions lasting approximately 40 min. The session consisted of completing two questionnaires and three experimental tasks.

Participants first completed the sociodemographic questionnaire, which included items on clinical history and early caregiving practices, followed by the Experience in Close Relationships Scale (ECR), adapted and validated in each country. Participants then performed the screen-based experimental tasks: (1) Ebbinghaus size comparison task, (2) Müller-Lyer line length comparison task, and (3) Personal Space Visual Task.

Before each task, participants completed three practice trials to ensure task comprehension. The total administration time for the experimental tasks was approximately 15 min (5 min per task).

Responses to the ECR questionnaire were scored according to the authors’ instructions. Experimental tasks were scored using three indices: the Ebbinghaus Accuracy Index (EAI), the Müller-Lyer Accuracy Index (MAI), and the Personal Space Distance Remaining Mean Index (PS-DR_m).

### 2.4 Data analysis

Prior to testing the study hypotheses, the distribution of all main variables was examined using the Kolmogorov–Smirnov test. Because several variables deviated from normality, non-parametric methods were used for bivariate group comparisons and correlational analyses. However, multivariate models were retained to adjust for country and gender, as regression assumptions concern primarily the distribution and behavior of residuals rather than the normality of raw variables.

Group comparisons were conducted using Chi-square tests for categorical variables (e.g., co-sleeping and co-bathing frequencies), with adjusted standardized residuals for *post-hoc* analyses. For continuous variables, Kruskal-Wallis tests were applied, followed by pairwise Mann–Whitney U tests with Bonferroni correction. Associations among child-rearing practices, attachment dimensions, and personal space regulation were examined using Spearman correlations, both in the full sample and stratified by country.

For all OLS models, regression diagnostics were examined, including residual distribution, homoscedasticity, multicollinearity, independence of errors, and influential observations. Where assumptions were not fully met, OLS findings were interpreted cautiously and considered alongside the non-parametric and quantile regression analyses.

To test Hypothesis 1, associations between early child-rearing practices (co-sleeping and co-bathing, coded as binary variables) and attachment-related avoidance and anxiety were examined. Initial comparisons were performed using Mann–Whitney U tests, followed by ordinary least squares (OLS) regression models including country and gender as covariates.

For Hypothesis 2, we investigated whether co-sleeping and co-bathing were related to tolerance for interpersonal closeness in the 2D screen-based task. Group differences were tested with Mann–Whitney U analyses and effect sizes (r) were calculated. To account for possible gender-specific associations, analyses were repeated separately for men and women. In addition, Ordinary Least Squares (OLS) regression models with interaction terms were estimated to assess whether the relationship between child-rearing practices and personal space tolerance varied across countries.

Finally, for Hypothesis 3, we tested whether attachment-related avoidance was associated with greater tolerance to interpersonal closeness in the 2D screen-based task. Bivariate associations were first examined using Spearman correlations. To complement these analyses and address distributional skewness, quantile regression models at the median (50th percentile) were also estimated, including country and gender as covariates.

## 3 Results

### 3.1 Descriptive analyses

Before testing the study hypotheses, we examined the distribution of child-rearing practices (co-sleeping and co-bathing), self-reported attachment-related avoidance and anxiety across participants’ country and gender.

The results showed significant differences in early child-rearing practices among Spain, Italy, and Japan. A chi-Square test revealed significant differences in co-sleeping practices frequencies across countries (χ2 = 44.55, *p* < 0.01). While Spain (79%) and Italy (56.47%) reported similar co-sleeping frequencies, Japanese participants (95%) reported a markedly higher frequency. *Post-hoc* analysis using adjusted standardized residuals showed that Japanese participants reported significantly higher rates of co-sleeping than expected, while Italian participants reported significantly lower rates. No significant difference was observed between Spain and Italy (Table 2). Similarly, a chi-square test for co-bathing confirmed significant cross-cultural differences (χ^2^ = 96.51, *p* < 0.01). Spain (36%) and Italy (35.29%) displayed comparable rates, whereas Japan (92.5%) reported substantially higher levels of co-bathing. *Post-hoc* comparisons using adjusted standardized residuals indicated that Japanese participants reported significantly higher rates of co-bathing than expected, whereas both Spanish and Italian participants reported significantly lower rates. Again, no significant difference was observed between Spain and Italy (Table 2).

**Table 2:** Co-sleeping and co-bathing frequencies for country with *post-hoc* analysis.

**Country**	**Co-sleep**			**Co-bath**		
	**Frequency**	**Chi-square (Pearson)**	Adj. residual *post-hoc* (p)*	**Frequency**	**Chi-square (Pearson)**	Adj. residual *post-hoc* (p)*
Japan	95%	χ^2^ = 44.55, *p* < 0.01	+2.0 (0.05)	92.5%	χ^2^ = 96.51, *p* < 0.01	+5.0 (<0.01)
Italy	56.50%		−2.3 (0.02)	35.30%		−2.9 (<0.01)
Spain	79%		0.0 (1.00)	36%		−2.8 (<0.01)

For clarity, frequencies and residuals are presented together; detailed χ^2^ values are reported in the text. *Adjusted standardized residuals greater than ±1.96 were considered statistically significant (*p* < 0.05).

Moreover, significant cross-country differences were observed in the duration of child-rearing practices engagement (Table 3). In Spain, the mean reported ending of co-sleeping age was 6.31 years (SD = 3.36), while in Japan it was slightly lower, at 5.66 years (SD = 3.69), and in Italy, it was 2.12 years (SD = 3.21). A Kruskal-Wallis test revealed a statistically significant difference in co-sleeping duration across countries, *H*(2) = 69.54, *p* < 0.01. *Post-hoc* pairwise comparisons using Mann–Whitney *U* tests with Bonferroni correction (*α* = 0.017) showed that Italy significantly differed from both Spain (*U* = 996.50, *p* < 0.01) and Japan (*U* = 2336.50, *p* < 0.01), while no significant difference was found between Spain and Japan (*U* = 3716.00, *p* = 0.03, n.s. after Bonferroni correction).

**Table 3:** Co-sleeping and co-bathing duration for countries with *post-hoc* analysis.

**Variable**	**Country**	**Duration Mean (SD)**	**Kruskal–Wallis**	Pairwise Group Differences (Mann–Whitney)*	
Co-sleeping	Japan	5.66 (3.69)	*H*(2) = 69.54, *p* < 0.01	Japan vs. Italy	*U* = 2336.50, *Z* = −6.75, *p* < 0.01
	Italy	2.12 (3.21)		Japan vs. Spain	*U* = 3716.00, *Z* = −2.19, *p* = 0.03
	Spain	6.31 (3.36)		Italy vs. Spain	*U* = 996.50, *Z* = −7.71, *p* < 0.01
Co-Bathing	Japan	5.47 (3.67)	*H*(2) = 79.29, *p* < 0.01	Japan vs. Italy	U = 1800.50, *Z* = −8.21, *p* < 0.01
	Italy	1.18 (2.20)		Japan vs. Spain	*U* = 2001.00, *Z* = −0.43, *p* = 0.67
	Spain	5.49 (2.64)		Italy vs. Spain	*U* = 331.00, *Z* = −7.23, *p* < 0.01

*Pairwise comparisons were conducted using Mann–Whitney U tests. A Bonferroni correction was applied to control for multiple comparisons, setting the significance threshold at *p* < 0.017 (0.05/3). Comparisons with *p*-values above this threshold are considered non-significant.

For co-bathing, Japanese participants reported the longest practice duration, with a mean end age of 5.47 years (SD = 3.67). In Spain, the practice ended at a similar age (*M* = 5.49, SD = 2.64), whereas in Italy the practice ended much earlier, at 1.18 years (SD = 2.20). This difference was also confirmed by a Kruskal-Wallis test, *H*(2) = 79.29, *p* < 0.01. *Post-hoc* pairwise comparisons using Mann–Whitney *U* tests with Bonferroni correction (*α* = 0.017) showed that Italy significantly differed from both Spain (*U* = 331.00, *p* < 0.01) and Japan (*U* = 1800.50, *p* < 0.01), while the difference between Spain and Japan was not significant (*U* = 2001.00, *p* = 0.67).

We also examined whether self-reported attachment-related avoidance and anxiety (measured with the ECR questionnaire) varied across countries and gender. Kruskal–Wallis tests revealed significant cross-country differences in attachment-related avoidance (*H* = 45.13, *p* < 0.01; Table 4), with Japanese participants reporting the highest score. Smaller but significant cross-country differences were also found for attachment-related anxiety, *H*(2) = 7.57, *p* = 0.02, with Italian participants reporting slightly higher anxiety scores than Spanish and Japanese participants.

**Table 4:** Descriptive Statistics and Kruskal-Wallis test results for attachment anxiety and avoidance by country and gender.

**Variable**	**Mean (SD)**		**Kruskal–Wallis (*p*-value)**
Anxiety (ECR-R)	Spain 61.18 (15.61)	Male 61.10 (16.93)	7.57 (*p* = 0.02)
		Female 61.26 (14.34)	
	Italy 63.26 (20.58)	Male 62.77 (16.25)	
		Female 63.47 (22.35)	
	Japan 55.4 (21.10)	Male 55.27 (19.38)	
		Female 55.69 (24.96)	
Avoidance (ECR-R)	Spain: 54.19 (14.61)	Male 54.62 (12.80)	45.13 (*p* < 0.01)
		Female 53.76 (16.34)	
	Italy: 47.78 (15.66)	Male 52.23 (12.91)	
		Female 45.81 (16.46)	
	Japan: 64.47 (18.71)	Male 65.95 (17.30)	
		Female 61.03 (21.52)	

To better understand the role of gender in these patterns, non-parametric Kruskal-Wallis tests were conducted separately by gender. Findings showed that attachment-related avoidance differed significantly across countries for both men (*H* = 22.36, *p* < 0.001) and women (*H* = 14.53, *p* < 0.001). In both groups, Japanese participants reported the highest levels of avoidance. In contrast, attachment-related anxiety did not significantly differ across countries in either men (*H* = 3.38, *p* = 0.185) or women (*H* = 2.93, *p* = 0.232).

Regarding personal distance perception in the 2D screen-based task, a Kruskal-Wallis test revealed significant differences across countries in the average distance at which participants allowed the humanoid avatar to approach before indicating discomfort (PS-DR_m scores; *H* = 39.02, *p* < 0.01; Table 5). This variable reflects the average remaining distance when participants stopped the avatar, with higher scores indicating lower tolerance for interpersonal closeness in the screen-based task. *Post-hoc* Mann–Whitney *U* tests identified specific differences between countries (Table 5). Spanish participants scored significantly higher than both Italian (*U* = 5482.0, *p* < 0.01) and Japanese participants (*U* = 8865.0, *p* < 0.01). Italian participants also scored significantly higher than Japanese participants (*U* = 6269.0, *p* = 0.01). In sum, Spanish and Italian participants stopped the avatar at a greater distance than Japanese participants, indicating lower tolerance to interpersonal closeness in the 2D screen-based task. No significant gender differences were found within each country.

**Table 5:** Differences in personal distance measure (PS-DR_m) across countries.

Mean (SD)		Kruskal–Wallis	Mann–Whitney - *post-hoc*	
Spain	8.42 (1.35)	39.02 (*p* < 0.01)	Spain vs. Italy	5482.0 (*p* < 0.01)
Italy	7.72 (1.60)		Spain vs. Japan	8865.0 (*p* < 0.01)
Japan	7.34 (1.31)		Italy vs. Japan	6269.0 (*p* < 0.01)

Results from the illusion task showed significant cross-cultural differences in susceptibility to optical illusions in the Ebbinghaus (EAI) and Müller-Lyer (MAI) indices (Table 6).

**Table 6:** Differences in illusion susceptibility across countries.

Variable	Country	Mean (DT)	Kruskal–Wallis	Mann–Whitney - *post-hoc*	
(EAI) Ebbinghaus Accuracy Index	Spain	−0.04 (1.54)	9.23 (*p* < 0.01)	Spain vs. Italy	5,260 (*p* = 0.02)
	Italy	−0.67 (1.37)		Spain vs. Japan	7135.5 (*p* = 0.04)
	Japan	−0.57 (1.47)		Italy vs. Japan	4813.5 (*p* = 1)
(MAI) Müller–Lyer Accuracy Index	Spain	45.31 (12.46)	17.79 (*p* < 0.01)	Spain vs. Italy	5729.5 (*p* < 0.01)
	Italy	40.00 (6.35)		Spain vs. Japan	6,794 (*p* = 0.27)
	Japan	42.62 (5.89)		Italy vs. Japan	3887.5 (*p* = 0.01)

Spanish participants were more susceptible to the Müller-Lyer illusion (*M* = 45.31, SD = 12.46), indicating greater distortion in visual–spatial judgment compared to Italian (*M* = 40.00, SD = 6.35) and Japanese participants (*M* = 42.62, SD = 5.89). In contrast, Italians were most susceptible to the Ebbinghaus illusion (*M* = −0.67, SD = 1.37), followed by Japanese (*M* = −0.57, SD = 1.47), while Spanish participants were least affected (*M* = −0.04, SD = 1.54).

To explore the relationship between visual processing and interpersonal space regulation, Spearman correlation analyses were conducted. In the Spanish sample, a significant negative correlation was found between MAI and PS-DR_m (*ρ* = −0.31, *p* < 0.01), indicating that greater susceptibility to the Müller-Lyer illusion was associated with shorter stopping distances in the 2D personal space task. In the Italian sample, the same trend was observed but did not reach statistical significance (ρ = −0.18, *p* = 0.09). No meaningful associations were found between susceptibility to the Ebbinghaus illusion (EAI) and personal distance perception in either country.

In contrast, in the Japanese sample, no significant correlations were observed between PS-DR_m (the average distance remaining with respect to the observer when stopping the avatar) and either illusion index (MAI: ρ = 0.07, *p* = 0.46; EAI: ρ = −0.02, *p* = 0.81), suggesting that in this cultural group, personal space regulation may rely less on low-level visual-perceptual mechanisms.

*H1*: Child rearing practices (co-bathing and co-sleeping) are associated with lower levels of self-reported attachment-related avoidance in adulthood.

The first hypothesis analysis examined whether child-rearing practices, specifically co-sleeping and co-bathing, were associated with self-reported attachment-related avoidance, assessed through the Experiences in Close Relationships Scale (ECR). The results did not support this hypothesis. Associations between early child-rearing practices and adult attachment-related avoidance were therefore examined using both bivariate and multivariate analyses. A series of Mann–Whitney U tests were conducted to compare levels of attachment-related avoidance and anxiety between participants reporting early caregiving co-sleeping or co-bathing experiences and those who had not (Table 7). Initial analyses revealed that co-sleeping and co-bathing practices were associated with attachment-related avoidance, statistically significant though small in magnitude (Table 7).

**Table 7:** Mann–Whitney U test results: child-rearing practices (co-sleeping and co-bathing) and attachment dimensions.

**ECR Dimension**	**Child-rearing practice**	***U***	***p*-value**	**Effect Size (*r*)**
Attachment-related Anxiety	Co-sleeping	7109.0	0.34	0.05
	Co-bath	9680.0	0.11	0.04
Attachment-related Avoidance	Co-sleeping	8705.5	0.03*	0.12
	Co-bath	13288.5	<0.01*	0.15

*Statistically significant results at *p* < 0.05.

However, when controlling for country and gender through OLS regression analyses, these associations were no longer significant. Regression diagnostics for the H1 OLS models indicated low multicollinearity and acceptable independence of errors. However, residual normality and homoscedasticity were not fully met in all models; therefore, OLS findings were interpreted cautiously and considered alongside the non-parametric analyses. In the model predicting attachment avoidance, neither co-bathing (*β* = −1.98, *p* = 0.41) nor co-sleeping (*β* = −1.37, *p* = 0.59) reached significance. Similarly, in the model predicting attachment anxiety, neither co-bathing (*β* = −2.06, p = 0.46) nor co-sleeping (*β* = 0.59, *p* = 0.85) emerged as significant predictors. Although initial bivariate analyses suggested significant links, these effects were not robust after adjusting for covariates.

In the OLS regression model predicting attachment-related avoidance, country remained a significant predictor after controlling for the other variables included in the model. Using Spain as a reference category, Japanese participants reported significantly higher levels of attachment-related avoidance (*β* = 10.88, *p* < 0.01) whereas Italian participants reported significantly lower levels (*β* = −6.02, *p* = 0.02). A marginal gender effect was also observed, with male participants tending to report higher avoidance than females (*β* = 3.72, *p* = 0.06).

To further refine these findings, we explored whether the duration of early caregiving practices (as measured by the reported age at which co-sleeping or co-bathing practices ended) was associated with adult self-reported attachment measures (Table 8). Using Spearman correlations, we found that longer duration of co-bathing was significantly related to higher attachment-related avoidance, and a similar, though weaker effect was observed for co-sleeping. No significant associations were found with attachment-related anxiety for either practice (Table 8).

**Table 8:** Spearman correlations for attachment practices and child-rearing practices duration.

ECR dimension	**End of co-sleep age**	**End of co-bath age**
Attachment-related Anxiety	−0.03 (*p* = 0.65)	−0.05 (*p* = 0.35)
Avoidance	0.12 (*p* = 0.04) *	0.17 (*p* < 0.01) *

*Statistically significant results at *p* < 0.05.

*H2*: Child-rearing practices (co-bathing and co-sleeping) are related to greater tolerance for interpersonal closeness in a 2D screen-based task.

The second hypothesis examined whether child-rearing practices (co-bathing and co-sleeping) were associated with tolerance for interpersonal closeness in the 2D screen-based task. The Mann–Whitney *U* test (Table 9) indicated that co-sleeping was more consistently associated with increased tolerance to closeness across multiple humanoid figures (all avatars except the boy and girl figures), whereas co-bathing primarily affected responses to the adult male figure, with only a tendency observed for the adult female figure (Table 9) in interpersonal distance measures. At the aggregate level (PS-DR_m_), co-sleeping was associated with shorter remaining distances (*U* = 6,086, *p* < 0.01, *r* = 0.15) although the effect size was small.

**Table 9:** Mann–Whitney *U* test results: differences in personal space based on co-sleeping and co-bathing practices.

**Variable**	**Group**	***U***	***p*-value**	Effect size **(*r*)**
PS-DR_m	Co-sleeping	6,086	<0.01*	0.15
	Co-bath	10,476	0.26	0.06
PS-DR-ROBOT	Co-sleeping	6,320	0.03*	0.13
	Co-bath	10,324	0.18	0.07
PS-DR-LBOY	Co-sleeping	6966.5	0.23	0.07
	Co-bath	10429.5	0.24	0.07
PS-DR-GIRL	Co-sleeping	6,817	0.15	0.08
	Co-bath	11,160	0.82	0.01
PS-DR-AMALE	Co-sleeping	6,331	0.03*	0.13
	Co-bath	9,462	0.01*	0.14
PS-DR-AFEMALE	Co-sleeping	6,285	0.02*	0.13
	Co-bath	9917.5	0.06	0.11
PS-DR-OFEMALE	Co-sleeping	6,409	0.04*	0.12
	Co-bath	10,799	0.49	0.04

*Statistically significant results at *p* < 0.05.

Gender-stratified analyses revealed different patterns of association between child-rearing practices and personal space measures (Table 10). Among women, co-sleeping was significantly associated with greater tolerance to closeness in the 2D screen-based task across multiple humanoid figures (PS-DR_m, *U* = 1485.0, *p* = 0.04) and, more specifically, with proximity to the robot figure (PS-DR-ROBOT, *U* = 1468.0, *p* = 0.03). Among men, co-bathing showed a stronger association, with significant effects on proximity to both the robot (*U* = 2137.0, *p* < 0.01) and the adult male figure (PS-DR-AMALE, *U* = 2056.0, *p* < 0.01). In addition, both co-sleeping (*U* = 1437.0, *p* = 0.04) and co-bathing (*U* = 2056.0, *p* < 0.01) were linked to men’s tolerance to closeness with the adult male figure. These gender-stratified findings should be interpreted cautiously given the small effect sizes and the exploratory nature of these subgroup analyses.

**Table 10:** Mann–Whitney *U* test results: differences in personal space based on co-sleeping and co-bathing practices by gender.

**Variable**	**Gender**	**Group**	***U***	***p*-value**	**Effect size (*r*)**
PS-DR_m	Female	Co-sleeping	1,485	0.04*	0.17
		Co-bath	2,636	0.97	<0.01
	Male	Co-sleeping	1,501	0.08	0.14
		Co-bath	2,406	0.06	0.15
PS-DR-ROBOT	Female	Co-sleeping	1,468	0.03*	0.18
		Co-bath	2,788	0.53	0.05
	Male	Co-sleeping	1,654	0.28	0.09
		Co-bath	2,137	<0.01*	0.22
PS-DR-LBOY	Female	Co-sleeping	1,670	0.24	0.10
		Co-bath	2,503	0.63	0.04
	Male	Co-sleeping	1771	0.57	0.04
		Co-bath	2,528	0.15	0.11
PS-DR-GIRL	Female	Co-sleeping	1,654	0.21	0.10
		Co-bath	2,647	0.94	0.01
	Male	Co-sleeping	1,696	0.37	0.07
		Co-bath	2,752	0.51	0.05
PS-DR-AMALE	Female	Co-sleeping	1,669	0.24	0.10
		Co-bath	2,483	0.57	0.05
	Male	Co-sleeping	1,437	0.04*	0.16
		Co-bath	2056	<0.01*	0.25
PS-DR-AFEMALE	Female	Co-sleeping	1,525	0.06	0.15
		Co-bath	24,155	0.40	0.07
	Male	Co-sleeping	1,596	0.18	0.11
		Co-bath	2,388	0.05*	0.15
PS-DR-OFEMALE	Female	Co-sleeping	1,583	0.11	0.13
		Co-bath	2,700	0.77	0.02
	Male	Co-sleeping	1,572	0.15	0.11
		Co-bath	2,522	0.14	0.12

*Statistically significant results at *p* < 0.05.

To examine whether the relationship between co-sleeping and personal space preferences (PS-DR_m) varied by country, an ordinary least squares regression (OLS) regression model with interaction terms was conducted. Regression diagnostics indicated acceptable independence of errors and no evidence of heteroscedasticity. However, residuals deviated from normality, and elevated multicollinearity was observed in the interaction terms involving country and caregiving practices, reflecting the uneven distribution of co-sleeping and co-bathing across countries. Accordingly, the interaction models were interpreted cautiously. The model explained 10.4% of the variance in personal space (*R*^2^ = 0.10), indicating that the associations observed at the bivariate level were no longer evident once country was controlled for. Co-sleeping did not show a significant main effect on tolerance to closeness in the 2D screen-based task (*β* = −0.47, *p* = 0.13), and the interaction between co-sleeping and country was also non-significant.

A parallel OLS regression analysis was conducted to assess whether co-bathing was associated with personal space preferences and whether this effect varied by country. The model explained 11.5% of the variance in personal space (*R*^2^ = 0.12). Co-bathing showed a marginal effect (*β* = 0.60, *p* = 0.06), indicating a trend toward significance but not reaching conventional thresholds. The interaction between co-bathing and country was also non-significant (*p* > 0.05), suggesting no cross-country variation in this association.

*H3*: Higher attachment-related avoidance is related to greater tolerance for interpersonal closeness in a screen-based task.

The third hypothesis examined whether higher attachment-related avoidance was associated with greater tolerance for interpersonal closeness in the 2D screen-based task.

Spearman’s rank-order correlations showed a significant negative association between attachment-related avoidance and personal space distance remaining scores (PS-DR_m; *ρ* = −0.17, *p* < 0.01), indicating that higher avoidance was linked to stopping the avatar at a shorter distance (i.e., greater tolerance for interpersonal closeness) in the 2D task. By contrast, attachment-related anxiety was not significantly correlated with closeness tolerance (ρ = 0.05, *p* = 0.38).

A quantile regression analysis at the median (50th percentile), including avoidance, anxiety, country, and gender as covariates, revealed that neither avoidance nor anxiety significantly predicted interpersonal closeness tolerance in the 2D screen-based task. In the same model, gender and country emerged as significant predictors of personal space tolerance: men showed greater tolerance for closeness than women (*β* = 0.42, *p* = 0.04). Using Spain as the reference category, Japanese participants showed significantly greater tolerance for closeness (*β* = −1.16, *p* < 0.01), and Italian participants also differed significantly from the Spanish group (*β* = −0.65, *p* = 0.01). Spaniards were the least tolerant, stopping the avatar at the largest remaining distance (Table 11).

**Table 11:** Quantile regression analysis predicting tolerance for interpersonal closeness in the 2D screen-based task (PS-DR_m index), with Spain as the reference category.

**Predictor**	***β***	***p*-value**
Attachment avoidance (ECR)	−0.08	0.27
Attachment anxiety (ECR)	0.05	0.38
Gender (Men vs. Women)	0.42*	0.04
Japan (vs. Spain, ref.)	−1.16*	< 0.01
Italy (vs. Spain, ref.)	−0.65*	0.01
Spain (reference)	0	---

*Statistically significant results at *p* < 0.05.

**Figure 4:**
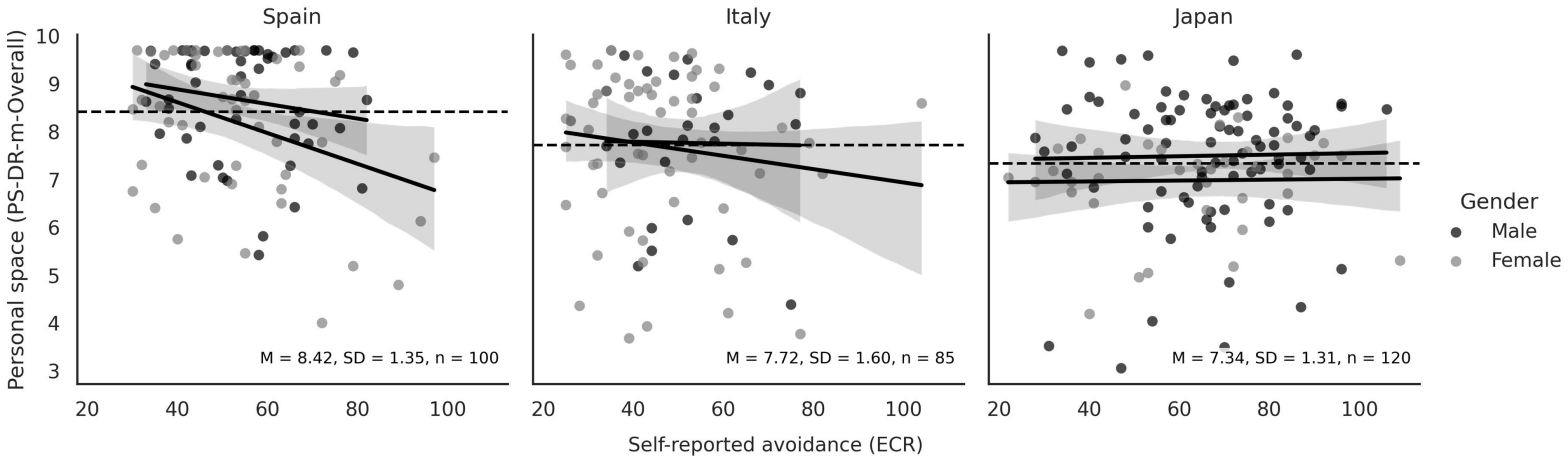
Relationship between attachment-related avoidance and personal space tolerance, stratified by country and gender.

At the multivariate level, attachment-related avoidance was not a significant predictor of personal space tolerance once country and gender were included (Figure 4).

## 4 Discussion

The present study examined the interplay between early child-rearing practices (co-sleeping and co-bathing), self-reported attachment avoidance and anxiety, and perceptual susceptibility to visual illusions in relation to interpersonal distance regulation in a virtual 2D screen-based task across Spanish, Italian, and Japanese contexts. Drawing on cross-cultural and social-personality approaches to adult attachment, the study examined whether retrospective reports of early caregiving practices were associated with adult attachment-related dimensions, particularly attachment-related avoidance (H1). These practices were also expected to be associated with individual differences in interpersonal distance regulation, influencing tolerance for interpersonal closeness in the virtual paradigm (H2). Finally, it was anticipated that higher levels of attachment-related avoidance would be linked to greater acceptance of interpersonal closeness within the 2D task (H3).

These hypotheses are grounded in the idea that higher attachment-related avoidance is associated with reduced emotional engagement and deactivating strategies in situations involving interpersonal closeness (Brennan et al., 1998; Mikulincer and Shaver, 2008, 2019). Unlike face-to-face encounters with attachment figures, virtual interactions with unfamiliar others may not strongly activate the attachment system, potentially enabling individuals with higher attachment-related avoidance to tolerate reduced interpersonal space without perceiving it as threatening (Cartaud et al., 2020; Riem et al., 2019). Furthermore, cultural norms and early caregiving practices such as co-sleeping and co-bathing are likely to shape perceptions of proximity, fostering cross-cultural variation in how avoidance is expressed (Esposito et al., 2015; McKenna et al., 2007; Yang and Hahn, 2002). In this context, the 2D paradigm provides a low-intensity social setting in which higher levels of attachment-related avoidance may be associated with a preference for autonomy. This preference may paradoxically manifest as greater tolerance for interpersonal closeness, reflecting deactivation strategies and the reduced emotional demands of virtual interactions (Han et al., 2023; Kroczek et al., 2020; Mikulincer and Shaver, 2023).

The study sample provides evidence of consistent cross-cultural differences between Spain, Italy, and Japan in early child-rearing practices, self-reported attachment dimensions, susceptibility to visual illusions, and tolerance for interpersonal closeness in a 2D screen-based task, offering the contextual background against which the hypotheses were tested.

Japanese participants reported substantially higher rates and longer durations of co-sleeping and co-bathing experiences compared to their Mediterranean counterparts, whereas Spain and Italy showed more similarities, with Italy standing out for the shortest durations. This aligns with prior research showing that co-sleeping is deeply normative in Japan and associated with fostering interdependence and emotional closeness (Esposito et al., 2015; Mileva-Seitz et al., 2017; Mindell et al., 2010a, 2010b; Yang and Hahn, 2002), whereas Mediterranean cultures adopt a more ambivalent stance (McKenna et al., 2007; Mileva-Seitz et al., 2017). More broadly, child-rearing practices should be understood as culturally embedded systems of value and meaning (Bornstein, 2019; Lansford, 2022, 2025; Lansford et al., 2021), which helps explain why the same caregiving behavior may foster relational security or closeness in one cultural context but be associated with overprotection in another. Moreover, retrospective reports of sleep-related practices are known to be shaped by cultural biases regarding what is perceived as normative caregiving (Mindell et al., 2013), a factor that may contribute to cross-country differences in the self-reported prevalence of co-sleeping and co-bathing.

Japanese participants reported the highest levels of attachment-related avoidance, whereas Italian participants reported the lowest, suggesting that avoidance is particularly sensitive to cultural orientations toward relationships. Although attachment-related anxiety also showed significant cross-cultural variation, these differences were smaller in magnitude than those observed for avoidance and did not systematically predict interpersonal distance once contextual factors were considered. These cultural variations are consistent with large-scale findings indicating higher attachment avoidance in Asian contexts and relatively higher attachment anxiety in Mediterranean groups (Bassi et al., 2022; Koeneke-Hoenicka et al., 2022; Nakao and Kato, 2004; Schmitt et al., 2004).

Japanese participants tolerated closer distances in the 2D screen-based task, whereas Spanish and Italian participants required greater interpersonal space. This finding partly challenges traditional assumptions of Mediterranean interpersonal closeness, unless a distinction is made between in-person physical proximity and virtual, screen-mediated proximity (Beaulieu, 2004; Sorokowska et al., 2017; Sussman and Rosenfeld, 1982). Because the screen-based task reduced emotional intensity and embodied interpersonal cues, culturally shaped preferences for closeness may have been expressed differently than they would be in real-life interactions. In this sense, the findings suggest that interpersonal distance regulation is context-sensitive and may differ across physical and digitally mediated settings. Thus, the present results highlight the importance of considering both cultural norms and methodological settings (i.e., virtual vs. face-to-face) when interpreting proxemic behavior. Beyond the experimental paradigm, these findings also resonate with the growing role of screens, mobile devices, and social media in shaping interpersonal interactions. Virtual interactions often reduce the emotional intensity of face-to-face encounters, which may allow individuals with higher attachment-related avoidance to feel more comfortable engaging in closeness through a screen or other digitally mediated formats (Biocca et al., 2003; Gritti et al., 2023; Kreijns et al., 2022).

Spaniards were more susceptible to the Müller-Lyer illusion, Italians to the Ebbinghaus illusion, whereas Japanese participants showed lower susceptibility to both. These cultural differences can be interpreted as the combined result of neuroanatomical variability and culture-specific cognitive styles. At the neurobiological level, Schwarzkopf et al. (2011) demonstrated that individual differences in the surface area of the primary visual cortex (V1) predict susceptibility to these illusions, suggesting that structural variability in early visual areas underlies perceptual biases. Complementarily, Hedden et al. (2008) found that during visual tasks requiring the integration or suppression of contextual information, Western and East Asian participants recruited distinct neural networks: Westerners engaged regions linked to focal, object-based attention, whereas East Asians showed stronger activation in areas supporting contextual and holistic processing. This evidence indicates that cultural frameworks shape attentional control at the neural level, highlighting an interplay between individual differences and culturally embedded cognitive-perceptual styles.

*H1*: Child-rearing practices and adult attachment avoidance.

Hypothesis 1 proposed that early child-rearing practices, specifically co-sleeping and co-bathing, would be associated with lower levels of self-reported attachment-related avoidance in adulthood. Support for this hypothesis was limited and context dependent. At the bivariate level, the presence of co-sleeping and co-bathing was weakly associated with lower avoidance, but these effects disappeared once country and gender were controlled. Although initial bivariate analyses suggested significant links, these effects were not robust after adjusting for covariates, indicating that broader cultural context accounted for a larger proportion of variance. This pattern suggests that co-sleeping and co-bathing may function less as independent developmental predictors and more as culturally embedded caregiving practices whose meanings and relational implications are shaped by broader sociocultural norms. Thus, their developmental relevance should be interpreted within the cultural contexts in which these practices occur, rather than as direct or universal predictors of adult attachment-related orientations.

This attenuation aligns with prior cross-cultural research showing substantial variability in attachment patterns, with Asian samples often reporting higher avoidance (Koeneke-Hoenicka et al., 2022; Nakao and Kato, 2004; Schmitt et al., 2004). Importantly, the mere presence of co-sleeping and co-bathing was not a reliable predictor of adult attachment dimensions; however, their prolonged duration was modestly but significantly associated with higher avoidance. This suggests that extended bodily proximity in early caregiving may not uniformly foster lower attachment-related insecurity. Developmental studies emphasize that the same caregiving practice can promote autonomy in one cultural context but be experienced as intrusive or overprotective in another (Bornstein, 2013; Lansford et al., 2021). This reflects the fact that cultural values and socialization goals shape the meanings children and parents attribute to caregiving practices (Mindell et al., 2013). Overall, the results caution against assuming a simple linear relationship between early proximity and later attachment-related orientations in adulthood, underscoring the need to interpret child-rearing practices within broader cultural frameworks (Bornstein, 2019; Lansford et al., 2021; Schmitt et al., 2004).

*H2*: Child-rearing practices and tolerance for interpersonal closeness

Hypothesis 2 proposed that co-sleeping and co-bathing experiences would be associated with greater tolerance for interpersonal closeness in the 2D screen-based task. Support for this hypothesis was modest and context dependent. Co-sleeping was consistently, though weakly, linked to greater tolerance for closeness, with small effect sizes across several avatar conditions. Gender differences also emerged, with women’s tolerance more influenced by co-sleeping and men’s by co-bathing, suggesting potential gender-specific pathways. However, these associations disappeared once country was controlled, indicating that the influence of early caregiving practices on personal space preferences may operate primarily in interaction with broader cultural frameworks (Sorokowska et al., 2017). Although initial bivariate analyses suggested significant links, these effects were not robust after adjusting for covariates, again indicating that cultural influences accounted for a larger proportion of variance. This interpretation aligns with prior work showing that interpersonal distance is not a fixed individual trait but varies systematically with cultural norms and situational cues (Schmitt et al., 2004; Sorokowska et al., 2017).

Methodological factors may also account for the weak effects. The virtual 2D paradigm used in this study captures interpersonal distance regulation in a relatively abstract form, which may further attenuate individual differences compared to real-world interactions. Indeed, studies using immersive virtual reality show that digital environments dampen emotional responses and alter proxemic behavior relative to face-to-face contexts (Kroczek et al., 2022; Riem et al., 2019). In addition, psychophysiological research demonstrates that affective cues, such as facial expressions, strongly predict comfortable distance, highlighting the role of perceptual and affective integration in interpersonal space regulation (Cartaud et al., 2020, 2022). However, these findings are not directly transferable to the present task, which relied on simplified 2D avatars and did not include dynamic facial expressions or autonomic measures. Rather, they help to underscore that interpersonal distance regulation may depend on different processes depending on the richness of socio-emotional cues available in the interaction. Taken together, these findings suggest that early caregiving practices may contribute to interpersonal distance regulation, but their influence is likely moderated by cultural context and shaped by situational and affective cues.

*H3*: Attachment avoidance and tolerance for interpersonal closeness

Hypothesis 3 proposed that higher attachment-related avoidance would be associated with greater tolerance for interpersonal closeness in the 2D screen-based task. At the bivariate level, this prediction received initial support, as avoidance correlated with shorter stopping distances of the avatar, indicating greater closeness tolerance in a virtual environment. However, this effect disappeared once country and gender were included in multivariate models, suggesting that cultural norms and demographic factors exert a stronger influence on interpersonal space regulation than individual attachment tendencies. Thus, although the initial bivariate association was consistent with the hypothesis, these effects were not robust after adjusting for covariates, suggesting that cultural context accounted for a larger proportion of variance in interpersonal distance regulation. This pattern further suggests that part of the initially observed association between attachment-related avoidance and closeness tolerance may reflect broader sociocultural and demographic influences shared across these variables. In this sense, attachment-related avoidance may be partly embedded within culturally patterned norms regarding interpersonal closeness and social interaction, which could explain why country emerged as a stronger and more stable predictor in the multivariate models.

This discrepancy may partly reflect the specific characteristics of the screen-based paradigm. In a 2D screen-based context, higher levels of attachment-related avoidance or anxiety may be associated with perceiving interpersonal proximity as less threatening, thereby attenuating the influence of attachment dimensions on personal space preferences (Kroczek et al., 2022; Riem et al., 2019). One possible interpretation is that, in this low-emotional context, visual proximity did not carry the same relational meaning as physical closeness in face-to-face interactions. The approaching avatars did not require direct reciprocity, involve physical vulnerability, or imply emotionally meaningful engagement. Consequently, individuals with higher attachment-related avoidance may have tolerated shorter distances because the task did not strongly activate the discomfort or defensive distancing usually associated with emotionally salient interpersonal closeness. More broadly, these findings are consistent with psychophysiological evidence showing that proxemic behavior is shaped primarily by perceptual and contextual cues, such as facial expressions or visual illusions, rather than by attachment alone (Bunce et al., 2021; Cartaud et al., 2022). Together, these results suggest that while attachment-related avoidance may be associated with closeness tolerance at the bivariate level, its influence is substantially moderated by cultural and situational determinants in virtual settings.

### 4.1 Limitations and future directions

Several limitations should be acknowledged when interpreting the present findings. First, the study employed a cross-sectional design, which prevents causal inferences about the developmental pathways linking child-rearing practices, attachment, perceptual processing, and personal space regulation. Accordingly, longitudinal approaches are needed to clarify how these associations unfold over time. Second, attachment was assessed through self-report measures, which may be subject to social desirability and cultural response biases. Incorporating complementary methods, such as observational or interview-based assessments, would provide a more robust evaluation. Third, the samples showed uneven gender distributions across countries, and consisted primarily of young university students, which may limit the generalizability of the findings to broader age and socioeconomic groups despite the use of statistical controls. Additionally, the reliance on retrospective self-reports of caregiving practices introduces recall bias, as adult participants’ reports of co-sleeping and co-bathing may be influenced by autobiographical reconstruction, cultural norms regarding normative caregiving, or imperfect childhood memory. Such measurement error may have attenuated the observed associations. Socioeconomic background was also not considered, although it may shape both child-rearing practices and personal space norms.

A further limitation concerns the reliance on a 2D screen-based paradigm to measure personal space. While this approach offers high standardization and replicability, prior evidence shows that virtual distance tasks often elicit attenuated emotional responses compared to face-to-face interactions (Kroczek et al., 2020, 2022; Riem et al., 2019). Moreover, the present task relied on simplified 2D avatars and did not include dynamic facial expressions, emotion-recognition demands, or autonomic measures, all of which may play an important role in interpersonal distance regulation in more naturalistic settings. Thus, future research should combine such paradigms with immersive or mixed-reality methodologies and multimethod assessments to capture more ecologically valid proxemic behavior.

A further limitation is that sociocultural influences were operationalized through country-level group comparisons rather than through direct measures of specific cultural values or norms. Accordingly, country should be interpreted as a pragmatic proxy for broader sociocultural environments rather than as a direct measure of culture itself. The observed cross-country differences may therefore also reflect other contextual factors, such as recruitment setting, demographic composition, or unmeasured social and educational differences across samples. Future research would benefit from including explicit sociocultural indicators—such as individualism–collectivism, family interdependence, or norms regarding interpersonal closeness—to better clarify the mechanisms underlying cross-cultural differences in interpersonal distance regulation.

Finally, although three distinct cultural groups were compared, the generalizability of the findings remains limited. Future studies should extend this line of inquiry to other cultural contexts (e.g., Northern Europe, Latin America, North America) and examine how perceptual sensitivity, attachment orientations, and cultural learning interact in shaping interpersonal space, ideally through multi-method and cross-cultural designs. It would also be valuable to examine how these processes differ across face-to-face, immersive virtual, and low-emotional screen-based contexts.

Addressing these limitations will allow future research to move beyond isolated explanations and toward integrative frameworks. Taken together, our results support a biopsychosocial-cultural perspective in which early caregiving, attachment dispositions, cultural norms, and perceptual processes jointly shape interpersonal distance. More specifically, the findings suggest that interpersonal distance regulation in a controlled screen-based context is more strongly structured by cultural norms and perceptual tendencies than by early caregiving practices or attachment dispositions alone. Future work would benefit from representing these interactions through conceptual or graphical models, which could guide more integrative theorizing and empirical testing.

## 5 Conclusion

The present study examined how child-rearing practices, attachment orientations, and perceptual processing are associated with interpersonal distance regulation across Spanish, Italian, and Japanese samples. While some bivariate associations emerged (such as links between co-sleeping and attachment-related avoidance or between attachment-related avoidance and closeness tolerance in a 2D screen-based task) these effects proved modest and disappeared once cultural and demographic factors were considered. Importantly, the mere presence of co-sleeping and co-bathing was not a reliable predictor of adult attachment dimensions; however, their prolonged duration was modestly but significantly associated with higher avoidance, suggesting that extended early bodily proximity may not uniformly promote lower attachment-related insecurity and may, in some contexts, be associated with overprotective dynamics rather than relational security. Instead, cross-cultural patterns in caregiving norms, attachment tendencies, and proxemic preferences accounted for much of the variance.

These findings highlight the need to situate developmental and relational experiences within their cultural frameworks. Cultural norms appear to exert a stronger influence than individual attachment dispositions on interpersonal space regulation, particularly in digitally mediated contexts. At the same time, these conclusions should be interpreted with reference to the methodological context of the study, as proximity preferences expressed in a controlled 2D screen-based task may not directly correspond to face-to-face interpersonal behavior. The results also indicate that the same caregiving practices can carry different meanings across cultural settings, fostering autonomy and security in some cases, but potentially overprotection or ambivalence in others. This underlines the importance of understanding early proximity experiences as embedded in systems of cultural values and socialization goals.

The integration of visual illusion tasks further demonstrates that perceptual mechanisms intersect with social behavior, with cultural differences in illusion susceptibility shaping how individuals regulate interpersonal space. This suggests that low-level perceptual processes can bias social judgments, while also being shaped by culturally embedded attentional and cognitive styles.

Finally, the present study shows that interpersonal distance regulation emerges from the dynamic interplay of attachment, child-rearing, perceptual, and cultural factors rather than from any single domain. Future research should therefore adopt longitudinal, cross-cultural, and ecologically valid approaches, including immersive virtual reality, dynamic socio-emotional cues, and physiological measures, to capture how these factors interact over time. By bridging cognitive, developmental, cultural, and technological perspectives, the field can move toward a more comprehensive biopsychosocial-cultural model of interpersonal distance regulation. From an integrative perspective, the present findings support a context-sensitive understanding of interpersonal distance regulation, in which cultural, perceptual, and relational factors interact within specific social and methodological settings.

## Data Availability

The datasets presented in this study can be found in online repositories. The names of the repository/repositories and accession number(s) can be found in the article/Supplementary material.
