# Supplementary material for: Cultural and relational factors in interpersonal distance regulation: evidence from a 2D screen-based task in Spain, Italy, and Japan
Source: Front Psychol. 2026 Jun 30;17:1802960. doi: 10.3389/fpsyg.2026.1802960 (PMC13364631; doi:10.3389/fpsyg.2026.1802960)
Supplement: Supplementary file 1 [file Table_1.DOCX]

**S1 - Sociodemographic and Received Upbringing Questionnaire**

| *This questionnaire is subject to strict professional confidentiality.* |
| --- |

| Subject ID (numeric): |  |
| --- | --- |
| Date of administration: |  |
| Time: |  |

1. **Country of birth: ________________________________**
2. **Country of residence: ________________________________**
3. **Number of years in Spain/Italy/Japan: ________________________________**
4. **Number of years of university education: ________________________________**
5. **What year are you in at the university?**

□ First

□ Second

□ Third

□ Fourth

□ Fifth

□ Other (specify): **________________________________**

**6. Occupation/profession:**

□ Student

□ Full-time worker

□ Other (specify): **________________________________**

**7. Gender:**

□ Male

□ Female

□ Prefer not to say

□ Other (specify): **________________________________**

**8. Date of birth:** **________________________________**

**9. Number of siblings:** **________________________________**

□ How many older than you? **________________________________**

□ And younger than you? **________________________________**

**10. Parents’ status:**

□ Alive and together

□ Alive and separated

□ Mother deceased when you were _____ years old

□ Father deceased when you were _____ years old

**11. Do you currently have a romantic relationship?**

□ Yes

□ No

**12. If you answered yes, how long have you been in that relationship?**

□ 0–6 months

□ 7–12 months

□ 13–18 months

□ 19–24 months

□ More than 25 months

**13. Have you ever suffered from a psychopathological disorder?**

□ Yes

□ No

**14. If you answered yes, what was the diagnosis? ________________________________**

**15. Are you currently taking any medication or receiving medical treatment in a hospital?**

□ Yes

□ No

**16. Do you have children?**

□ Yes

□ No

**17. Have you ever practiced co-sleeping with your parents? (***That is, did you sleep with your parents during childhood?***)**

□ Yes

□ No

**18. ANSWER ONLY IF QUESTION 17 WAS ANSWERED AFFIRMATIVELY**

**18.1 How many times per week did you sleep with your parents? ___________________**

**18.2 How long per night?**

□ All night (You went to bed directly with them)

□ Part of the night (You went to bed in your own bed and then went to your parents’ bed)

**18.3 When did you stop bedsharing? Please answer in terms of “age.” _______________**

**19. Have you ever bathed with one of your parents?**

□ Yes

□ No

**20. ANSWER ONLY IF QUESTION 19 WAS ANSWERED AFFIRMATIVELY**

**20.1 What were the baths like?**

□ We bathed together

□ He bathed me

□ She bathed me

**18.3 When did you stop doing it? Please answer in terms of “age.” _______________**
